# Supplementary material for: Pharmacist involved education program in a multidisciplinary team for oral mucositis: Its impact in head-and-neck cancer patients
Source: PLoS One. 2021 Nov 22;16(11):e0260026. doi: 10.1371/journal.pone.0260026 (PMC8608342; doi:10.1371/journal.pone.0260026)
Supplement: S1 Dataset — (PDF) [file pone.0260026.s001.pdf]

No	age	gender	weight1	weight2	weight char	cancer grade1	cancer grade2
1	56	1	59.5	55.0	(4.5)	0	0
2	61	1	60.5	57.1	(3.4)	0	0
3	77	1	61.6	59.2	(2.4)	0	0
4	74	1	62.9	54.5	(8.4)	0	1
5	64	1	60.9	60.1	(0.8)	0	1
6	61	1	65.8	61.5	(4.3)	0	0
7	68	1	65.3	61.7	(3.6)	0	0
8	54	1	57.8	52.5	(5.3)	0	0
9	72	1	41.2	36.7	(4.5)	0	0
10	64	1	85.4	79.8	(5.6)		1
11	72	1	51.8	45.4	(6.4)	0	0
12	52	1	63.5	56.6	(6.9)	0	1
13	67	1	71.0	68.0	(3.0)	0	0
14	70	2	39.0	36.3	(2.7)	0	1
15	59	1	68.0	60.8	(7.2)	0	0
16	70	1	57.9	55.3	(2.6)	0	0
17	73	1	65.4	61.1	(4.3)	0	0
18	69	1	60.7	59.0	(1.7)	0	0
19	60	1	58.5	57.0	(1.5)	0	0
20	59	1	64.4	56.3	(8.1)	0	0
21	59	1	68.8	61.6	(7.2)	0	0
22	61	1	66.9	63.4	(3.5)	0	1
23	74	1	60.6	58.7	(1.9)	0	0
24	67	1	60.5	59.4	(1.1)	0	0
25	66	1	56.9	52.1	(4.8)	0	0
26	29	1	61.9	53.9	(8.0)	0	1
27	64	1	52.2	49.2	(3.0)	0	0
28	60	1	60.2	54.7	(5.5)	0	0
29	47	1	83.9	78.6	(5.3)	0	0
30	48	1	56.4	54.3	(2.1)	0	0
31	64	1	81.0	75.1	(5.9)	0	0
32	56	2	45.3	43.2	(2.1)	0	0
33	67	1	60.5	61.5	1.0	0	0
34	78	1	59.5	57.3	(2.2)	0	0
35	77	1	61.3	58.0	(3.3)	0	0
36	77	1	70.7	64.2	(6.5)	0	0
37	78	1	64.9	61.3	(3.6)	0	0
38	48	1	75.2	70.2	(5.0)	0	0
39	74	1	52.9	52.7	(0.2)	0	0
40	64	1	54.4	50.5	(3.9)	0	1
41	77	1	58.3	57.4	(0.9)	0	0
42	56	1	97.7	93.5	(4.2)	0	0
43	55	1	63.6	60.5	(3.1)	0	0
44	62	1	53.3	51.0	(2.3)	0	0
45	51	1	74.3	69.4	(4.9)	1	0
46	46	1	60.9	59.1	(1.8)	1	0
47	77	1	66.9	61.4	(5.5)	0	1
48	50	2	54.4	55.4	1.0	0	0

49	60	1	70.5	64.8	(5.7)	0	1
50	61	1	58.0	55.7	(2.3)	0	0
51	79	1	53.5	50.2	(3.3)	1	0
52	60	1	48.5	48.4	(0.1)	0	1
53	77	1	71.0	69.7	(1.3)	0	0

cancer grade3	cancer grade4	sodiumu azulene	dimethlyllisopropy	Gy	switching to fentanyl tape	Alb1	Alb2
1	0	80	60	70	1	3.7	3.9
1	0	40	80	70	0	4.0	3.3
1	0	50	160	70	0	3.9	3.1
0	0	95	380	70	1	4.2	3.6
0	0	30	20	70	0	3.4	3.3
0	1	35	180	70	1	3.7	3.8
0	1	45	300	70	0	4.0	3.9
0	1	105	140	60	1	2.4	2.6
0	1	25	20	70	1	3.7	3.3
		15	60	70	0	4.2	4.2
1	0	100	60	70	1	3.5	3.3
0	0	90	20	70	1	3.6	3.8
1	0	70	260	70	0	3.6	3.1
0	0	80	120	70	1	4.1	3.9
1	0	65	100	70	1	4.1	4.1
1	0	100	220	70	1	4.8	–
0	1	80	180	70	0	3.4	–
0	1	35	200	70	1	3.9	3.7
0	1	95	40	70	0	3.5	3.5
0	1	100	180	70	0	4.1	3.7
0	1	25	100	70	0	3.7	3.2
0	0	50	100	70	0	4.0	4.3
1	0	25	60	70	1	3.6	3.6
1	0	60	120	70	1	4.2	4.1
0	1	65	380	70	0	3.5	3.6
0	0	40	60	60	0	4.6	3.8
1	0	70	180	70	1	3.8	3.4
0	1	65	100	70	0	4.0	3.4
1	0	60	220	70	1	3.7	3.7
0	1	50	160	70	1	3.9	3.7
1	0	90	160	70	1	4.3	3.8
1	0	25	20	66	0	2.8	3.4
1	0	40	180	70	0	4.0	4.0
1	0	150	220	70	0	3.6	2.8
0	1	25	120	68	1	3.4	3.7
0	1	95	220	70	1	3.5	3.3
0	1	0	80	70	0	4.0	3.7
0	1	80	60	70	0	4.1	3.7
0	1	20	20	70	0	3.7	–
0	0	45	100	70	0	3.6	3.2
0	1	75	0	70	0	3.6	3.4
1	0	40	160	60	0	3.8	3.3
0	1	65	280	70	0	4.2	3.5
0	1	120	300	70	0	4.2	3.8
0	0	55	140	70	1	3.9	3.6
0	0	20	120	70	0	4.0	3.6
0	0	85	60	70	1	4.2	3.6
0	1	0	80	70	0	3.5	4.0

0	0	45	180	70	0	4.0	3.6
1	0	15	60	70	0	3.2	3.2
0	0	30	120	70	0	4.1	4.1
0	0	25	60	70	0	4.0	3.6
0	1	60	200	70	0	2.9	2.5

Alb3	Alb4	Alb median	WBC1	WBC2	WBC3	WBC4	WBCmedian	Neu1
3.7	3.5	3.7	7000.0	5290.0	4140.0	4250.0	4770.0	5170.0
3.3	3.3	3.3	6870.0	5300.0	4560.0	3250.0	4930.0	–
3.3	3.4	3.4	5660.0	4000.0	2740.0	3470.0	3735.0	3850.0
3.4	3.1	3.5	5430.0	3450.0	2090.0	3860.0	3655.0	3210.0
3.4	3.3	3.4	5870.0	4270.0	6450.0	4500.0	5185.0	2900.0
3.2	3.7	3.7	5860.0	6830.0	5390.0	4430.0	5625.0	3730.0
3,3	3,7	4.0	4990.0	3580.0	2540.0	3360.0	3470.0	3010.0
2.5	2.5	2.5	13730.0	9080.0	11470.0	2970.0	10275.0	11190.0
2.8	2.3	3.1	5800.0	4790.0	6050.0	1190.0	5295.0	3540.0
3.9	–	4.2	6340.0	7130.0	4880.0	4350.0	5610.0	4380.0
3.3	3.4	3.4	5110.0	4770.0	2440.0	1540.0	3605.0	2650.0
3.2	3.6	3.6	7040.0	6770.0	4830.0	4910.0	5840.0	4450.0
3.6	3.3	3.5	6380.0	4930.0	4380.0	3550.0	4655.0	3920.0
3.2	3.2	3.6	5560.0	5500.0	3920.0	2460.0	4710.0	3600.0
4.0	3.5	4.1	5940.0	8800.0	6330.0	6570.0	6450.0	2950.0
3.6	4.1	4.1	4770.0	4490.0	2740.0	3820.0	4155.0	3620.0
–	3.4	3.4	5150.0	7110.0	5010.0	2480.0	5080.0	3570.0
3.5	3.2	3.6	6470.0	3420.0	5540.0	2410.0	4480.0	4050.0
3.9	3.6	3.6	2520.0	1840.0	2130.0	4500.0	2325.0	1350.0
3.8	3.7	3.8	5270.0	9490.0	4900.0	4790.0	5085.0	4280.0
3.0	3.5	3.4	8150.0	8450.0	4800.0	4670.0	6475.0	5760.0
3.7	4.2	4.1	4780.0	3700.0	4250.0	3770.0	4010.0	3000.0
3.2	3.3	3.5	3790.0	5100.0	4870.0	3000.0	4330.0	2020.0
3.4	4.1	4.1	5960.0	5000.0	4270.0	5810.0	5405.0	3930.0
3.7	3.5	3.6	7930.0	3710.0	3680.0	3260.0	3695.0	6780.0
4.0	4.1	4.1	6060.0	6380.0	5330.0	3150.0	5695.0	3850.0
3.5	3.7	3.6	4300.0	3990.0	4680.0	4830.0	4490.0	2810.0
3.5	2.9	3.5	6530.0	5170.0	3840.0	2890.0	4505.0	3810.0
3.8	3.6	3.7	10670.0	5770.0	4040.0	2490.0	4905.0	8070.0
3.9	4.2	3.9	7870.0	5700.0	3690.0	2980.0	4695.0	5250.0
3.8	3.9	3.9	4230.0	4050.0	2350.0	2110.0	3200.0	2420.0
3.4	3.4	3.4	9930.0	5310.0	5070.0	3450.0	5190.0	6640.0
3.8	3.9	4.0	3710.0	3110.0	4850.0	4020.0	3865.0	2030.0
3.1	3.0	3.1	7780.0	4770.0	5110.0	3220.0	4940.0	5710.0
4.0	2.9	3.6	7990.0	5720.0	7380.0	2770.0	6550.0	7200.0
3.7	3.6	3.6	7860.0	5500.0	8300.0	5590.0	6725.0	5960.0
3.5	3.7	3.7	5310.0	5600.0	2630.0	2380.0	3970.0	3160.0
3.8	3.7	3.8	5600.0	4160.0	2250.0	3060.0	3610.0	3530.0
–	–	3.7	3110.0	3740.0	3430.0	2600.0	3270.0	1460.0
3.7	3.5	3.6	14590.0	10310.0	6480.0	5160.0	8395.0	11330.0
3.6	3.4	3.5	2490.0	3130.0	5430.0	1970.0	2810.0	810.0
3.4	3.5	3.5	6130.0	6340.0	5740.0	3540.0	5935.0	4010.0
3.7	3.8	3.8	9230.0	9200.0	6650.0	2950.0	7925.0	5440.0
3.7	3.6	3.8	5310.0	3840.0	4060.0	1430.0	3950.0	4190.0
3.4	3.2	3.5	7310.0	6060.0	4870.0	3400.0	5465.0	4550.0
3.8	3.8	3.8	6370.0	4660.0	3860.0	3620.0	4260.0	3170.0
–	2.5	3.6	4130.0	4000.0	3160.0	2710.0	3580.0	2290.0
3.5	4.0	3.8	5240.0	4910.0	5710.0	5840.0	5475.0	3330.0

3.4	3.8	3.7	7870.0	5660.0	3480.0	2880.0	4570.0	5690.0
3.4	3.6	3.3	10320.0	5970.0	3670.0	3750.0	4860.0	7040.0
3.9	3.6	4.0	4810.0	4110.0	3290.0	3200.0	3700.0	3100.0
3.5	3.4	3.6	5220.0	2270.0	3050.0	2010.0	2660.0	4070.0
2.5	3.0	2.7	12390.0	8610.0	6270.0	4560.0	7440.0	9590.0

Neu2	Neu3	Neu4	Neumedian	PLT1	PLT2	PLT3	PLT4	PLTmedian	eGFR1
4270.0	3280.0	3520.0	3895.0	28.2	21.1	22.9	24.1	23.5	79.1
3870.0	3270.0	2500.0	3270.0	20.6	15.6	16.8	16.2	16.5	80.5
3340.0	2030.0	2720.0	3030.0	12.1	11.1	11.6	7.3	11.4	82.4
2430.0	1260.0	3080.0	2755.0	13.8	7.9	22.0	10.6	12.2	74.0
2860.0	5010.0	3310.0	3105.0	28.9	21.6	22.7	18.7	22.2	118.3
5000.0	3020.0	3510.0	3620.0	22.7	20.2	20.4	21.1	20.8	104.3
2710.0	1590.0	2900.0	2805.0	28.8	25.1	17.5	16.9	21.3	91.4
7810.0	10430.0	2190.0	9120.0	46.9	39.7	46.2	35.1	43.0	104.2
3960.0	5370.0	530.0	3750.0	21.3	11.4	18.6	15.8	17.2	79.0
5520.0	3680.0	3520.0	4030.0	18.5	19.2	17.3	19.4	18.9	87.3
3520.0	1590.0	1420.0	2120.0	11.6	7.6	10.2	5.8	8.9	74.6
5100.0	3840.0	4470.0	4460.0	23.1	27.1	12.0	15.3	19.2	105.3
4050.0	3600.0	2880.0	3760.0	24.7	21.7	17.5	22.6	22.2	80.3
4490.0	3080.0	1810.0	3340.0	23.9	15.3	19.4	18.2	18.8	101.1
7470.0	4910.0	5630.0	5270.0	25.2	21.1	38.0	15.1	23.2	64.4
3910.0	2070.0	3340.0	3480.0	17.5	13.2	11.6	9.8	12.4	74.9
5560.0	3780.0	1390.0	3675.0	26.8	15.8	16.8	11.1	16.3	71.0
2580.0	4500.0	1710.0	3315.0	31.6	18.1	25.7	20.4	23.1	82.4
1100.0	1180.0	3270.0	1265.0	19.1	15.2	19.9	11.2	17.2	95.5
8630.0	4450.0	3970.0	4365.0	27.1	29.2	35.5	23.2	28.2	101.6
6490.0	3490.0	3390.0	4625.0	31.4	29.8	28.7	20.6	29.3	98.1
2550.0	3350.0	3100.0	3050.0	19.7	13.6	16.6	14.9	15.8	93.9
3480.0	3810.0	1960.0	2750.0	19.8	18.3	17.1	12.5	17.7	73.0
3640.0	4270.0	4940.0	4100.0	23.8	19.1	8.8	20.5	19.8	88.5
3060.0	3130.0	2670.0	3095.0	14.7	10.3	12.6	10.9	11.8	76.5
5170.0	4080.0	2290.0	3965.0	44.2	30.1	22.1	20.9	26.1	98.2
3000.0	3930.0	3310.0	3155.0	23.1	23.7	30.6	45.8	27.2	76.1
4100.0	2820.0	2160.0	3315.0	31.4	22.3	22.3	24.0	23.2	87.1
4700.0	3090.0	2040.0	3895.0	25.5	24.2	15.2	18.4	21.3	90.7
4470.0	2840.0	2020.0	3655.0	29.8	17.8	12.2	12.0	15.0	101.2
3210.0	1760.0	1490.0	2090.0	18.7	12.1	13.9	9.4	13.0	81.8
3780.0	3050.0	2260.0	3415.0	19.1	14.1	24.4	16.0	17.6	79.4
2200.0	4190.0	3080.0	2640.0	15.1	6.2	8.8	8.6	8.7	88.5
3900.0	3670.0	2440.0	3785.0	30.4	18.1	33.3	24.0	27.2	60.2
4540.0	6100.0	1210.0	5320.0	23.6	24.4	25.5	18.0	24.0	111.7
3730.0	6330.0	3720.0	4845.0	28.3	29.2	26.7	37.3	28.8	83.6
4420.0	1770.0	1820.0	2490.0	23.7	18.5	17.7	12.3	18.1	74.0
3370.0	1680.0	2600.0	2985.0	25.5	17.0	15.7	13.8	16.4	79.4
2630.0	1980.0	1330.0	1720.0	13.6	16.9	18.9	17.6	17.3	75.4
7920.0	4420.0	4210.0	6170.0	33.8	24.4	39.6	21.9	29.1	67.6
2170.0	4490.0	1340.0	1755.0	11.7	8.2	11.3	6.5	9.8	71.2
4490.0	4030.0	2250.0	4020.0	24.9	19.5	21.5	21.8	21.7	99.6
6570.0	4850.0	1390.0	5145.0	17.9	13.4	15.8	10.5	14.6	99.6
3060.0	3450.0	830.0	3255.0	21.0	15.9	11.2	16.3	16.1	93.5
4350.0	3810.0	2140.0	4080.0	24.0	19.3	21.2	24.6	22.6	92.7
3340.0	2880.0	2380.0	3025.0	22.1	22.8	21.5	24.0	22.5	84.3
3180.0	2070.0	2110.0	2200.0	16.7	8.5	9.9	7.8	9.2	68.4
2870.0	3360.0	4310.0	3345.0	19.7	27.3	26.7	51.1	27.0	76.0

4280.0	2780.0	1880.0	3530.0	24.9	17.8	17.8	12.0	17.8	75.4
4350.0	2650.0	3170.0	3760.0	57.0	38.3	28.5	35.3	36.8	70.3
3190.0	2750.0	2550.0	2925.0	20.6	17.0	15.5	17.5	17.3	70.7
1690.0	2320.0	1500.0	2005.0	34.9	22.0	18.8	20.4	21.2	97.6
6910.0	4180.0	3300.0	5545.0	45.1	34.4	31.1	19.5	32.8	81.1

eGFR2	eGFR3	eGFR4	eGFRmedian	Scr1	Scr2	Scr3	Scr4	Scr median	CRP1	CRP2
58.2	69.1	71.7	70.4	0.8	1.0	0.9	0.9	0.9	0.1	0.1
73.1	59.0	62.4	67.7	0.8	0.8	1.0	1.0	0.9	－	0.1
85.0	59.3	67.5	74.9	0.7	0.7	0.7	0.8	0.7	－	1.1
70.1	61.8	93.5	72.1	0.8	0.8	0.9	0.6	0.8	0.0	0.1
118.3	105.2	101.5	111.8	0.5	0.5	0.6	0.6	0.6	0.1	0.1
75.1	85.4	85.4	85.4	0.6	0.8	0.9	0.7	0.8	－	1.2
80.7	69.0	76.9	78.8	0.7	0.7	0.9	0.8	0.8	2.0	0.1
116.4	100.6	114.2	109.2	0.6	0.6	0.6	0.6	0.6	21.9	3.4
73.6	54.5	101.3	76.3	0.7	0.8	1.0	0.6	0.8	0.1	－
67.1	59.7	58.8	63.4	0.7	0.9	1.0	1.0	1.0	0.1	0.2
79.0	81.4	71.6	76.8	0.8	0.7	0.7	0.8	0.8	1.4	－
95.2	85.5	88.1	91.7	0.6	0.7	0.8	0.7	0.7	0.1	0.1
72.8	41.6	64.1	68.5	0.7	0.8	1.4	0.9	0.9	0.1	0.5
81.1	78.0	96.4	88.8	0.5	0.6	0.6	0.5	0.6	0.1	0.3
51.2	44.8	62.2	56.7	0.9	1.2	1.3	1.0	1.1	0.2	2.8
66.5	65.7	55.5	66.1	0.8	0.9	0.9	0.9	0.9	0.0	－
72.0	64.1	57.7	67.6	0.8	0.8	0.9	1.0	0.9	3.2	1.5
87.8	87.8	83.7	85.8	0.7	0.7	0.7	0.7	0.7	－	0.5
86.7	78.3	97.2	91.1	0.7	0.7	0.8	0.6	0.7	0.0	0.1
107.2	103.4	115.8	105.3	0.6	0.6	0.6	0.6	0.6	0.0	5.4
86.2	80.1	84.9	85.6	0.6	0.7	0.8	0.7	0.7	－	－
79.4	82.9	86.7	84.8	0.7	0.8	0.7	0.7	0.7	0.0	0.0
79.6	88.9	83.3	81.5	0.8	0,7	0,7	0.7	0.8	7.0	0.5
62.8	43.6	73.1	68.0	0.7	0.9	1.3	0.8	0.9	1.4	0.0
74.4	68.8	68.8	71.6	0.8	0.8	0.9	0.9	0.9	0.0	0.3
94.2	94.2	109.0	96.2	0.8	0.8	0.8	0.7	0.8	0.1	0.0
75.1	54.6	68.5	71.8	0.8	0.8	1.1	0.9	0.9	0.6	7.4
76.5	67.2	96.0	81.8	0.7	0.8	0.9	0.7	0.8	0.1	0.2
93.5	78.8	96.4	92.1	0.7	0.7	0.8	0.7	0.7	－	0.0
88.8	79.4	68.3	84.1	0.7	0.7	0.8	0.9	0.8	0.4	0.2
77.2	86.9	80.6	81.2	0.7	0.8	0.7	0.8	0.8	0.1	0.4
90.4	88.6	80.4	84.5	0.6	0.5	5.0	0.6	0.6	－	0.1
84.4	96.2	87.1	87.8	0.7	0.7	0.6	0.7	0.7	0.0	0.0
65.5	67.2	56.2	62.9	0.9	0.9	1.2	1.0	1.0	0.4	0.3
78.7	70.2	114.0	95.2	0.5	0.7	0.8	0.5	0.6	0.1	0.4
86.4	83.7	72.2	83.7	0.7	0.7	0.7	0.8	0.7	0.4	6.4
70.0	67.2	66.4	68.6	0.8	0.8	0.8	0.8	0.8	0.1	0.1
95.9	86.2	94.4	90.3	0.8	0.7	0.8	0.7	0.8	0.1	0.0
42.9	46.0	50.0	48.0	0.8	1.3	1.2	1.1	1.2	－	－
70.9	57.9	52.2	62.8	0.9	0.8	1.0	1.1	1.0	1.8	5.0
85.0	64.9	79.9	75.6	0.8	0.7	0.9	0.7	0.8	0.1	0.0
88.9	82.5	93.2	91.1	0.6	0.7	0.8	0.7	0.7	0.3	0.7
82.5	80.2	94.7	88.6	0.6	0.8	0.8	0.7	0.8	0.5	2.4
79.0	66.6	51.4	72.8	0.7	0.8	0.9	1.1	0.9	0.0	0.0
86.0	82.4	83.5	84.8	0.7	0.8	0.8	0.8	0.8	0.1	0.1
82.0	83.2	81.0	82.6	0.8	0.8	0.8	0.8	0.8	0.0	－
67.5	49.3	77.5	68.0	0.8	0.8	1.1	0.7	0.8	0.1	1.1
83.1	81.6	78.7	80.2	0.6	0.6	0.6	0.6	0.6	0.1	0.0

61.3	60.6	61.3		61.3	0.8	1.0	1.0	1.0		1.0	0.0	0.2
76.1	74.1	66.1		72.2	0.9	0.8	0.8	0.9		0.9	0.1	0.1
68.8	66.1	81.8		69.8	0.8	0.8	0.9	0.7		0.8	0.8	0.0
88.5	101.1	88.5		93.1	0.6	0.7	0.6	0.7		0.7	0.1	－
66.6	73.2	68.4		70.8	0.7	0.9	0.8	0.8		0.8	12.5	6.5

CRP3	CRP4	CRP median	steroid	immunosupressant	oral care drug1
0.5	1.8	0.3	0	0	1
0.1	0.3	0.1	0	0	1
－	0.3	0.7	0	0	1
0.2	2.7	0.2	0	0	1
0.3	4.8	0.2	0	0	1
0.4	3.9	1.2	0	0	1
0.2	1.0	0.6	0	0	1
1.1	10.4	6.9	0	0	1
－	－	0.1	0	0	1
0.1	－	0.1	0	0	1
0.8	1.8	1.4	0	0	1
0.1	1.2	0.1	0	0	1
0.1	0.9	0.3	0	0	1
0.1	0.8	0.2	0	0	1
3.6	－	2.8	0	0	1
－	0.8	0.4	0	0	1
－	11.7	3.2	0	0	1
0.2	3.5	0.5	0	0	1
0.0	0.1	0.1	0	0	1
1.3	1.6	1.5	0	0	1
－	－	－	0	0	1
0.1	0.1	0.1	0	0	1
0.1	0.3	0.4	0	0	0
2.1	0.1	0.8	0	0	1
0.3	0.3	0.3	0	0	1
0.0	0.1	0.1	0	0	1
1.9	1.0	1.5	0	0	1
0.5	2.4	0.4	0	0	1
0.0	0.1	0.0	0	0	1
0.2	0.1	0.2	0	0	1
1.3	0.4	0.4	0	0	1
0.1	0.0	0.1	0	0	1
0.5	0.1	0.1	0	0	1
1.1	0.3	0.4	0	0	1
－	－	0.3	0	0	1
0.6	0.3	0.5	1	0	1
0.0	0.1	0.1	0	0	1
0.0	0.0	0.0	0	0	1
－	－	－	0	0	1
0.1	0.0	1.0	0	0	1
0.7	2.1	0.4	0	0	1
0.4	0.5	0.5	0	0	1
0.1	0.6	0.6	0	0	1
0.3	1.1	0.2	0	0	1
0.1	0.2	0.1	0	0	1
－	－	0.0	0	0	1
－	12.3	1.1	0	0	1
0.1	0.1	0.1	0	0	1

0.1	0.7	0.2	0	0	1
0.1	0.1	0.1	0	0	1
0.3	0.4	0.4	0	0	1
−	−	0.1	0	0	1
0.2	0.6	3.6	0	0	1

oral care drug12	oral care drug13	oral care drug14	oral care drug5	oral mucosite
1	0	0	0	1
1	0	0	0	1
1	0	0	0	1
1	1	0	0	1
1	0	0	0	1
1	0	0	0	1
1	0	0	0	1
1	1	0	0	1
1	0	0	0	0
1	0	0	0	0
1	1	0	0	1
1	0	0	0	1
1	0	0	0	1
1	0	0	0	1
1	1	1	0	1
1	0	0	0	1
1	1	1	0	1
1	1	0	0	1
1	0	0	0	0
1	0	0	1	1
1	0	0	0	1
1	0	0	1	1
0	0	0	0	0
1	0	0	0	1
1	0	0	0	1
1	0	0	0	1
1	0	0	0	1
1	1	0	1	1
1	1	0	0	1
1	1	1	0	1
1	0	0	0	0
1	0	0	0	0
1	0	0	0	1
1	0	0	0	1
1	0	0	0	1
1	0	0	0	1
1	0	0	0	1
1	0	0	0	1
1	0	0	0	1
1	0	0	0	1
1	0	0	0	1
1	0	0	0	0
1	0	0	0	1
1	0	0	0	1
1	0	0	0	0
1	0	0	0	1
1	0	0	0	1
1	0	0	0	0

1	0	0	0	1
1	0	0	0	0
1	0	0	0	1
1	0	0	0	0
1	0	0	0	1

hospital day opioide		days until opioid use	oral opioide dose
67	1	19	2130
57	1	18	585
61	1	22	960
68	1	18	4170
59	1	24	870
102	1	21	1430
68	1	18	1050
64	–	–	–
102	1	19	2940
57	0	–	0
54	1	14	3285
58	1	15	1095
57	1	13	1245
81	1	15	2565
67	1	5	4470
58	1	23	1680
68	1	31	1860
69	1	13	4950
61	–	–	–
61	1	9	2400
54	–	–	–
66	1	16	2850
51	–	–	–
63	1	18	1335
55	1	31	435
68	0	–	0
69	1	18	2250
67	1	23	3675
54	1	17	2640
79	–	–	–
61	1	19	2760
61	0	–	0
54	1	22	495
75	1	18	2535
69	1	17	2415
67	1	33	1500
54	1	14	1425
54	0	–	0
51	0	–	0
64	–	–	–
62	1	20	1125
53	0	–	0
52	1	22	780
57	1	16	2400
66	1	17	2400
53	1	41	210
61	1	21	1815
52	0	–	0

54	1	21	1305
59	1	17	1080
55	1	18	1365
67	0	-	0
52	0	-	0

G1 OM days	G2 OM days	G3 OM days
15	-	-
20	34	-
29	38	-
23	23	-
20	35	-
18	18	49
16	23	-
21	21	21
-	-	-
-	-	-
25	25	-
17	31	-
28	28	-
37	37	-
10	23	-
16	23	46
18	18	-
15	15	-
-	-	-
27	41	-
17	17	-
28	28	-
-	-	-
29	29	29
25	25	-
14	14	-
23	-	-
23	37	37
17	38	-
7	17	-
15	24	-
-	-	-
21	21	-
39	39	-
29	29	29
14	14	28
14	-	-
33	33	
22	-	-
29	29	-
26	26	-
-	-	-
7	21	-
16	30	-
-	-	-
34	34	-
30	30	-
-	-	-

	31	–	–
–		–	–
	24	24	–
–		–	–
	15	27	–

G4 OM days	EPMT	
–	0	
–	0	
–	0	
–	0	
–	0	
–	0	
–	0	
–	0	
–	0	
–	0	
–	0	
–	0	
–	0	
–	0	
–	0	
–	0	
–	0	
–	0	
–	0	
–	0	
–	0	
–	0	
–	0	
39	0	
–	0	
–	0	
–	0	
–	0	
–	0	
–	0	
–	1	
–	1	
–	1	
–	1	
–	1	
–	1	エピシル
–	1	
–	1	
–	1	
–	1	
–	1	
–	1	
–	1	
–	1	
–	1	
–	1	
–	1	
–	1	

–	1
–	1
–	1
–	1
–	1
