# Supplementary material for: Pharmacist involved education program in a multidisciplinary team for oral mucositis: Its impact in head-and-neck cancer patients
Source: PLoS One. 2021 Nov 22;16(11):e0260026. doi: 10.1371/journal.pone.0260026 (PMC8608342; doi:10.1371/journal.pone.0260026)
Supplement: Attachment — Submitted filename: Response to Reviewers.docx [file pone.0260026.s003.docx]

The paper documented the clinical outcomes of a pharmacist-involved education intervention among patients with head and neck cancer receiving concurrent chemoradiotherapy.

Major issues:

- There is potential of selection bias with a retrospective study design. For example, what are the selection criteria for a patient to be enrolled in the intervention compared to those without? The authors need to specify the efforts to reduce this bias. Although baseline characteristics of the two groups showed no statistical significance, but how were the number of study subjects determined and chosen? By inclusion of all and so-happened both groups were equal? Unless it is shown that selection bias is adequately addressed, the results and conclusion of the study may be invalid.

→Thank you very much for your suggestion. Pharmacist was educated by a dentist in January 2018. Therefore, the surveillance period was chosen to compare the year prior to January 2018, when no education was received, and the year after January 2018, when education was received.

We considered that the following as a method to reduce selection bias.

・All patients who underwent CCRT during the investigation period were surveyed as a countermeasure against selection bias.

・Investigators were not involved CCRT as a countermeasure against medical surveillance bias.

・ Presence/absence of oral mucositis and grade evaluator are not all author.

It is an exploratory study, and there were no similar reports in the past, so sample size could not be calculated. Therefore, from the viewpoint of feasibility, we investigated two-year survey period and conducted a retrospective survey. With the publication of this paper, it will be possible to calculate the sample size in the future, so I would like to determine patient information in more detail and randomized controlled trials. Thank you for your wonderful suggestions.

We modified the manuscript as follows:

P5L6

This study was performed with the approval of the Research Ethics Niigata university Hospital (approval number 2018-0201). The subjects were patients who were undergoing CCRT from February 2017 to January 2019. All patients received a first cycle of chemotherapy. Pharmacist was educated by a dentist in January 2018. Therefore, the surveillance period was chosen to compare the year prior to January 2018, when no education was received, and the year after January 2018, when education was received. All patients received CCRT on a regimen of cisplatin 80 mg/m^2^ on days 1, 22, and 43 during the aforementioned time period.

A retrospective survey of electronic medical records was conducted with the cooperation of the Medical Information Department of Niigata university Hospital. The patient data collected included age, gender, weight loss, steroid or immunosuppressant use, hematological values (albumin, white blood cell count, blood platelets, and neutrophils), cancer grade, primary cancer site, type and use of mouthwash and moisturizer, opioid use (yes/no, days until the start of opioid use, and dose), switch to fentanyl tape, and LOD. All patients who underwent CCRT during the investigation period were surveyed as a countermeasure against selection bias. Investigators were not involved CCRT as a countermeasure against medical surveillance bias. Presence/absence of OM and grade evaluator are not all author.

P8L5

Patient background characteristics were summarized by group (PEMT: yes/no). The two groups were compared using Fisher's exact test for qualitative data and the Mann-Whitney U test for quantitative data, and a significance level of *p<0.05* was set. JMP^®^ 14.2 (SAS Institute Inc., Cary, NC, USA) was used for statistical analysis. It is an exploratory study, and there were no similar reports in the past, so sample size could not be calculated. Therefore, from the viewpoint of feasibility, we investigated two-year survey period and conducted a retrospective survey.

- An in-depth discussion of **how** a pharmacist helped in improving the clinical outcomes is required. Any documented increase in adherence to the prescribed regimen? It is suggested that more references to previous literatures should be included, especially in the field of oral care to substantiate the authors’ claims.
- →Thank you very much for your suggestion. This study was retrospective study, so there was no document. Therefore, we investigated increase in adherence by the amount of mouthwash and moisturizer used, but could not follow the detailed information and could not show the statistical difference. However, as the pharmacist explained, we always checked on a face-to-face basis once a week that moisturizing was performed after moisturizer and proper storage of mouthwash (in darkness) was followed. In the future, We would like to proceed with more detailed prospective research.

We added 4references to previous literatures. Please check again.

P12L14

In addition, randomised controlled study reported that mouth wash and education program promote better physical and quality of life in CCRT［14,15］. Mouthwash provides a moist environment for the oral mucosa and decreases pain, improves xerostomia and promotes oral comfort［16,17］. Therefore, it is very important to continue mouthwash properly.

Minor issues:

- Duplicate keywords. Suggest ‘patient education’ to replace ‘education program’, ‘education’ and ‘advanced dental education’

→Thank you for your suggestion. We corrected it.

P5L14　Patient education

- Please include statistical analysis in the abstract. Why 53 patients? Total samples during the stipulated timeframe?

→Thank you for your suggestion. We include statistical analysis in the abstract.

As you pointed out, there were 53 people during the stipulated timeframe.

We corrected it.

P2L7

Total samples data of 53 patients during the stipulated timeframe were retrospectively collected from electronic medical records from February 2017 to January 2019. We compared the presence/absence of OM (OM: yes/no) between patients with and without PEMT (PEMT: yes/no) as the primary endpoint and OM severity as the secondary endpoint.

The following information was surveyed: age, gender, weight loss, steroid or immunosuppressant use, hematological values (albumin, white blood cell count, blood platelets, and neutrophils), cancer grade, primary cancer site, type and use of mouthwash and moisturizer, opioid use (yes/no, days until the start of opioid use, and dose, switch to tape), and length of hospital day (LOD). The two groups were compared using Fisher's exact test for qualitative data and the Mann-Whitney U test for quantitative data, and a significance level of p<0.05 was set.

- Please include study setting in the abstract

→Thank you for your suggestion. We include study setting in the abstract. Same as the above correction.

- p-value should not be capitalized

→We corrected it.

- Table 1: please double check the gender ratio under non-PEMT

→Thank you very much for your suggestion. We corrected it. Please check again.

- Table 1: weight loss should be an outcome rather than baseline characteristic

→Thank you very much for your suggestion. We added Table.

Table 2. Weight loss

- Table 2: total sample was 53 but reduced to 47 in table 2?

→Thank you very much for your suggestion. Because patients who have already used opioids before the target period are not included summary of data for patients receiving opioids. Very sorry for the lack of explanation. We added manuscript.

P6L6

The opioid dose used was determined from the oral morphine conversion ratio of the Japanese Society for Palliative Medicine as follows: oral morphine: oral oxycodone: transdermal fentanyl = (30:20:1) ［12］. Patients who have already used opioids before the target period are not included summary of data for patients receiving opioids. The LOD was the period from the CCRT start date to discharge from the hospital.

- line 2 under introduction: amount

→Thank you very much for your suggestion. We corrected it. Please check again.

P4L3

Oral mucositis (OM) has been described as the most painful aspect of chemotherapy［1］. Due to increased pain attributed to OM, the ingested amount of food is decreased, motivation with respect to treatment is reduced, and treatment-related deaths can occur, all of which warrant a change in the therapeutic approach［2］.

- Please provide the full name of the statistical software under methodology

→Thank you for your suggestion. We corrected JMP^®^ 14.2 (SAS Institute Inc., Cary, NC, USA).

P8L5

Patient background characteristics were summarized by group (PEMT: yes/no). The two groups were compared using Fisher's exact test for qualitative data and the Mann-Whitney U test for quantitative data, and a significance level of *p<0.05* was set. JMP^®^ 14.2 (SAS Institute Inc., Cary, NC, USA) was used for statistical analysis.

- What are the criteria for PEMT compared to those without? If without PEMT, does it mean just without pharmacist (other healthcare professionals are available) or without a multidisciplinary team?

→Thank you very much for your suggestion. Pharmacist was educated by a dentist in January 2018. Therefore, the surveillance period was chosen to compare the year prior to January 2018, when no education was received, and the year after January 2018, when education was received. →Same as the above correction.

If without PEMT, it mean that pharmacist did not educate CCRT patient (other healthcare professionals are available).

- If this was not the patients’ first cycle of chemotherapy, what was the history of OM occurrence between both comparison groups?

→Thank you for your suggestion. All patients received a first cycle of chemotherapy. We added manuscript.

P5L6

This study was performed with the approval of the Research Ethics Niigata university Hospital (approval number 2018-0201). The subjects were patients who were undergoing CCRT from February 2017 to January 2019. All patients received a first cycle of chemotherapy.

- 5^th^ paragraph of discussion: how had the involvement of a pharmacist reduced pain?

→Thank you for your suggestion. The involvement of a pharmacist reduced lidocaine viscous, opioid doses, and the rate of fentanyl tape switching due to mouth pain. We considered that this result was attributed to the correct use of mouthwash and moisturizer to reduce the severity of OM.

P14L5

PEMT had a significant effect on reduction of weight loss, use of lidocaine viscous, opioid dose, and rate of switch to fentanyl tape.

Other

We corrected the manuscript according to the PLOS ONE guidelines. Please check again.

About the supplementary information, we did not see any need for it.

We would like to appreciate it if you could tell us what information you need.
