# Supplementary material for: Pharmacist involved education program in a multidisciplinary team for oral mucositis: Its impact in head-and-neck cancer patients
Source: PLoS One. 2021 Nov 22;16(11):e0260026. doi: 10.1371/journal.pone.0260026 (PMC8608342; doi:10.1371/journal.pone.0260026)
Supplement: Attachment — Submitted filename: Response to Reviewers.docx [file pone.0260026.s005.docx]

Thank you very much for the efforts in the latest revision. The article is now more convincing and reads better.

Just a few minor suggestions as below:

Thank you very much for carefully checking our paper. We were able to correct it to a better manuscript.

1. Please double check the affiliation superscripts on the title page. What’s the difference between:
   a. ¶: These authors contributed equally to this work.
   b. &: These authors also contributed equally to this work.

→Thank you very much for your suggestion. According to PLOS ONE's style requirements, we marked ¶ for the firstly contributed equally to this work and & for the secondly contributed equally to this work.

P1L21

^¶^: These authors contributed firstly equally to this work.

^&^: These authors also contributed secondly equally to this work.

2. Please ensure p-value is not capitalised in the main text and the figures

→Thank you very much for your suggestion. We corrected it.

P8L19

Of the 53 patients enrolled in this study, 23 patients received PEMT and 30 patients did not. The combination of SASH + sodium bicarbonate (NaHCO_3_) + lidocaine hydrochloride viscous was used by 0% of the patients who received PEMT versus 30% (9 patients; *p*<0.01 Table 1) of those who did not.

3. Please standardize to use either Figure or Fig.

→Thank you very much for your suggestion. We standardize to use Fig.

Fig1. Pharmacist-involved education program in the multidisciplinary team

4. Materials and methods> Subjects>
   a. Please capitalise ‘University Hospital’
   b. ‘the’ first cycle of chemotherapy
   c. >Definition of terms: please either define RT or use CCRT

→Thank you very much for your suggestion. We corrected it.

P5L6

This study was performed with the approval of the Research Ethics Niigata University Hospital (approval number 2018-0201). All data were fully anonymized, so the Research Ethics Niigata University Hospital waived the requirement for informed consent.

P5L17

A retrospective survey of electronic medical records was conducted with the cooperation of the Medical Information Department of Niigata University Hospital.

P5L9

All patients received the first cycle of chemotherapy. Pharmacist was educated by a dentist in January 2018.

P6L5

Weight loss was defined as the difference in body weight before the initiation of CCRT (0 Gy) and after the delivery of all fractions (70 Gy).

5. Results> Opioids> line 2 should be (Table 3)

→Thank you very much for your suggestion. We corrected it.

P10L5

Opioids were used by 65.2% of patients (15 cases) who received PEMT and by 92.0% (23 cases) of those who did not; although the difference between groups was not significant (Table 3).

6. There are differences between values in Table 3 and in the result paragraph. Please double check.

→Thank you very much for your suggestion. We corrected it.

P10L9

The median opioid dose was significantly different between groups; 1102.5 (0–1736.5) mg in the PEMT group and 1860.5 (1050.5–2850.0) mg in the non-PEMT group (*p<0.01*).

7. Definition of RT is not needed in Table 1 since the term isn’t there.

→Thank you very much for your suggestion. We deleted RT.

8. Please remove the unnecessary ‘ ) ’ after a superscript annotation in all tables

→Thank you very much for your suggestion. We deleted its, please check again.

9. Maybe can consider to the delete the intepretation of whisker plot?
   a. **Fig 3 Length of hospital days.** ~~a Data are shown in a box and whisker plot. The ends of the box represent the upper and lower quartiles and the median is depicted by the horizontal line within the box.~~

→Thank you very much for your suggestion. We deleted it.

P12L1

**Fig 3 Length of hospital days.**

Other

We corrected references.

P16L1

1. Bellm LA, Epstein JB, Rose-Ped A, Martin P, Fuchs HJ. Patient reports of complications of bone marrow transplantation. Support Care Cancer. 2000; 8: 33–39.
2. Keefe DM, Schubert MM, Elting LS, Sonis ST, Epstein JB, Raber-Durlacher JE, et al. Mucositis Study Section of the Multinational Association of Supportive Care in Cancer and the International Society for Oral Oncology. Updated clinical practice guidelines for the prevention and treatment of mucositis. Cancer 2007; 109: 820–831.
3. Elting LS, Cooksley CD, Chambers MS, Garden AS. Risk, outcomes, and costs of radiation-induced oral mucositis among patients with head-and-neck malignancies. Int J Radiat Oncol Biol Phys. 2007; 68: 1110–1120.
4. Peterson DE, Boers-Doets CB, Bensadoun RJ, Herrstedt J, ESMO Guidelines Committee. Management of oral and gastrointestinal mucosal injury: ESMO Clinical Practice Guidelines for diagnosis, treatment, and follow-up. Ann Oncol. 2015; 5: 139–151.
5. Elad S, Cheng KKF, Lalla RV, Yarom N, Hong C, Logan RM, et al. Mucositis Guidelines Leadership Group of the Multinational Association of Supportive Care in Cancer and International Society of Oral Oncology (MASCC/ISOO)(2020) MASCC/ISOO clinical practice guidelines for the management of mucositis secondary to cancer therapy. Cancer. 2020; 126: 4423–4431.
6. Saito H, Watanabe Y, Sato K, Ikawa H, Yoshida Y, Katakura A, et al. Effects of professional oral health care on reducing the risk of chemotherapy-induced oral mucositis. Support Care Cancer. 2014; 22: 2935–2940.
7. Kubota K, Kobayashi W, Sasaki H, Nakagawa H, Kon T, Mimura M, et al. Professional oral health care reduces oral mucositis pain in patients treated by superselective intra-arterial chemotherapy concurrent with radiotherapy for oral cancer. Support Care Cancer. 2015; 23: 3323–3329.
8. Legert KG, Remberger M, Ringden O, Heimdahl A, Dahllof G. Reduced intensity conditioning and oral care measures prevent oral mucositis and reduces days of hospitalization in allogeneic stem cell transplantation recipients. Support Care Cancer. 2014; 22: 2133–2140.
9. Soga Y, Sugiura Y, Takahashi K, Nishimoto H, Maeda Y, Tanimoto M, et al. Progress of oral care and reduction of oral mucositis--a pilot study in a hematopoietic stem cell transplantation ward. Support Care Cancer. 2010; 19: 303–307.
10. The European Oral Care in Cancer Group (EOCC). Oral care guidance 2018. Japanese Association of Supportive Care in Cancer Web site. http://jascc.jp/wp/wp-content/uploads/2018/01/guidance_20180115.pdf. Accessed February 20, 2020.
11. Leppla L, Geest SD, Fierz K, Deschler-Baier B, Koller A. An oral care self-management support protocol (OrCaSS) to reduce oral mucositis in hospitalized patients with acute myeloid leukemia and allogeneic hematopoietic stem cell transplantation: a randomized controlled pilot study. Support Care Cancer. 2016; 24: 773–782.
12. Japanese Society for Palliative Medicine. Japanese Society for Palliative Medicine Guidelines 2010. Japanese Society for Palliative Medicine Web site. www.jspm.ne.jp/guidelines/pain/2010/chapter02/02 04 01 05.php. Accessed February 20, 2020.
13. Yokota T, Tachibana H, Konishi T, Yurikusa T, Hamauchi S, Sakai K, et al. Multicenter phase II study of an oral care program for patients with head and neck cancer receiving chemoradiotherapy. Support Care Cancer. 2016; 24: 3029–3036.
14. Yüce UÖ, Yurtsever S. Effect of Education About Oral Mucositis Given to the Cancer Patients Having Chemotherapy on Life Quality. J Cancer Educ. 2019; 34: 35-40.
15. B-S Huang, S-C Wu, C-Y Lin, K-H Fan, J T-C Chang, S-C Chen. The effectiveness of a saline mouth rinse regimen and education programme on radiation-induced oral mucositis and quality of life in oral cavity cancer patients: A randomised controlled trial. Eur J Cancer Care (Engl). 2018; 27: e12819.
16. Dorothy M Keefe, Mark M Schubert, Linda S Elting, Stephen T Sonis, Joel B Epstein, Judith E Raber-Durlacher et al. Updated clinical practice guidelines for the prevention and treatment of mucositis. Cancer. 2007; 109: 820-831.
17. Edward B Rubenstein, Douglas E Peterson, Mark Schubert, Dorothy Keefe, Deborah McGuire, Joel Epstein. Clinical practice guidelines for the prevention and treatment of cancer therapy-induced oral and gastrointestinal mucositis. Cancer. 2004; 100: 2026-2046.
18. Okada H, Onda, Shoji, Kotani K, Nakayama T, Nakagawa Y, Sakane N. Effects of lifestyle intervention performed by community pharmacists on glycemic control in patients with type 2 diabetes: the community pharmacists assist (Compass) project, a pragmatic cluster randomized trial. Pharmacol Pharmacy. 2016; 7: 124–132.
19. Saito N, Imai Y, Muto T, Sairenchi T. Low body mass index as a risk factor of moderate to severe oral mucositis in oral cancer patients with radiotherapy. Support Care Cancer. 2012; 20: 3373–3377.
