# Supplementary material for: Pharmacist involved education program in a multidisciplinary team for oral mucositis: Its impact in head-and-neck cancer patients
Source: PLoS One. 2021 Nov 22;16(11):e0260026. doi: 10.1371/journal.pone.0260026 (PMC8608342; doi:10.1371/journal.pone.0260026)
Supplement: Attachment — Submitted filename: R3resonse to Reviewers.docx [file pone.0260026.s006.docx]

Reviewer #1: Table 2 and Table 3 > please delete the ‘)’ behind a superscript
→Thank you very much for your suggestion. We deleted its, please check again.

Page 10 and Table 3, Opioids> consider to delete the trailing zeros, 19 and 18
→Thank you very much for your suggestion. We deleted its, please check again.

P10

Opioids were used by 65.2% of patients (15 cases) who received PEMT and by 92% (23 cases) of those who did not; although the difference between groups was not significant (Table 3). Days until the start of opioid use were 19 (17–21.5) days in the PEMT group and 18 (15–21.5) days in the non-PEMT group, resulting in no significant difference between groups. The median opioid dose was significantly different between groups; 1102.5 (0–1736.3) mg in the PEMT group and 1860 (1050–2850) mg in the non-PEMT group (*p<0.01*). Oral oxycodone was switched to fentanyl tape in a significantly lower percentage of patients in the PEMT group (22.7%; 5 cases) than non-PEMT group (64.0%; 16 cases) (Table 3).

Table 3> Maybe need to provide a brief reason for the exclusion at footnote a and b (eg because patient(s) was/were on opioids before CCRT)
→Thank you very much for your suggestion. We added it, please check again.

The figures in Table 3 and result paragraph ‘Opioids’ are still different. 1860.5 in paragraph but 1860.0 in Table 3. 1736.5 in paragraph but 1736.3 in Table 3. Consider to remove all trailing zeros (eg 15, instead of 15.0)
→Thank you very much for checking carefully. We corrected its, please check again. Same as the above correction.

Please standardize references to use small caps for all the first alphabets of each word in the journal article/report title.
→Thank you very much for checking carefully. We corrected its.

1. Bellm LA, Epstein JB, Rose-Ped A, Martin P, Fuchs HJ. patient reports of complications of bone marrow transplantation. support care cancer. 2000; 8: 33–39.
2. Keefe DM, Schubert MM, Elting LS, Sonis ST, Epstein JB, Raber-Durlacher JE, et al. mucositis study section of the multinational association of supportive care in cancer and the international society for oral oncology. updated clinical practice guidelines for the prevention and treatment of mucositis. cancer 2007; 109: 820–831.
3. Elting LS, Cooksley CD, Chambers MS, Garden AS. risk, outcomes, and costs of radiation-induced oral mucositis among patients with head-and-neck malignancies. int j radiat oncol biol phys. 2007; 68: 1110–1120.
4. Peterson DE, Boers-Doets CB, Bensadoun RJ, Herrstedt J, ESMO Guidelines Committee. management of oral and gastrointestinal mucosal injury: ESMO clinical practice guidelines for diagnosis, treatment, and follow-up. ann oncol. 2015; 5: 139–151.
5. Elad S, Cheng KKF, Lalla RV, Yarom N, Hong C, Logan RM, et al. mucositis guidelines leadership group of the multinational association of supportive care in cancer and international society of oral oncology (MASCC/ISOO)(2020) MASCC/ISOO clinical practice guidelines for the management of mucositis secondary to cancer therapy. cancer. 2020; 126: 4423–4431.
6. Saito H, Watanabe Y, Sato K, Ikawa H, Yoshida Y, Katakura A, et al. effects of professional oral health care on reducing the risk of chemotherapy-induced oral mucositis. support care cancer. 2014; 22: 2935–2940.
7. Kubota K, Kobayashi W, Sasaki H, Nakagawa H, Kon T, Mimura M, et al. professional oral health care reduces oral mucositis pain in patients treated by superselective intra-arterial chemotherapy concurrent with radiotherapy for oral cancer. support care cancer. 2015; 23: 3323–3329.
8. Legert KG, Remberger M, Ringden O, Heimdahl A, Dahllof G. reduced intensity conditioning and oral care measures prevent oral mucositis and reduces days of hospitalization in allogeneic stem cell transplantation recipients. support care cancer. 2014; 22: 2133–2140.
9. Soga Y, Sugiura Y, Takahashi K, Nishimoto H, Maeda Y, Tanimoto M, et al. progress of oral care and reduction of oral mucositis-a pilot study in a hematopoietic stem cell transplantation ward. support care cancer. 2010; 19: 303–307.
10. The European Oral Care in Cancer Group (EOCC). Oral care guidance 2018. Japanese Association of Supportive Care in Cancer Web site. http://jascc.jp/wp/wp-content/uploads/2018/01/guidance_20180115.pdf. Accessed February 20, 2020.
11. Leppla L, Geest SD, Fierz K, Deschler-Baier B, Koller A. an oral care self-management support protocol (OrCaSS) to reduce oral mucositis in hospitalized patients with acute myeloid leukemia and allogeneic hematopoietic stem cell transplantation: a randomized controlled pilot study. support care cancer. 2016; 24: 773–782.
12. Japanese Society for Palliative Medicine. Japanese Society for Palliative Medicine Guidelines 2010. Japanese Society for Palliative Medicine Web site. www.jspm.ne.jp/guidelines/pain/2010/chapter02/02 04 01 05.php. Accessed February 20, 2020.
13. Yokota T, Tachibana H, Konishi T, Yurikusa T, Hamauchi S, Sakai K, et al. multicenter phase II study of an oral care program for patients with head and neck cancer receiving chemoradiotherapy. support care cancer. 2016; 24: 3029–3036.
14. Yüce UÖ, Yurtsever S. effect of education about oral mucositis given to the cancer patients having chemotherapy on life quality. j cancer educ. 2019; 34: 35-40.
15. B-S Huang, S-C Wu, C-Y Lin, K-H Fan, J T-C Chang, S-C Chen. the effectiveness of a saline mouth rinse regimen and education programme on radiation-induced oral mucositis and quality of life in oral cavity cancer patients: a randomised controlled trial. eur j cancer care (engl). 2018; 27: e12819.
16. Dorothy M Keefe, Mark M Schubert, Linda S Elting, Stephen T Sonis, Joel B Epstein, Judith E Raber-Durlacher et al. updated clinical practice guidelines for the prevention and treatment of mucositis. cancer. 2007; 109: 820-831.
17. Edward B Rubenstein, Douglas E Peterson, Mark Schubert, Dorothy Keefe, Deborah McGuire, Joel Epstein. clinical practice guidelines for the prevention and treatment of cancer therapy-induced oral and gastrointestinal mucositis. cancer. 2004; 100: 2026-2046.
18. Okada H, Onda, Shoji, Kotani K, Nakayama T, Nakagawa Y, Sakane N. effects of lifestyle intervention performed by community pharmacists on glycemic control in patients with type 2 diabetes: the community pharmacists assist (Compass) project, a pragmatic cluster randomized trial. pharmacol pharmacy. 2016; 7: 124–132.
19. Saito N, Imai Y, Muto T, Sairenchi T. low body mass index as a risk factor of moderate to severe oral mucositis in oral cancer patients with radiotherapy. support care cancer. 2012; 20: 3373–3377.

Discussion> Switching to fentanyl tape was significantly lower among those patients who received PEMT versus those who did not, possibly because those procedures reduced their oral pain.> Consider to elaborate this (eg. Enforcement of correct mouthwash and moisturizer use by pharmacists enables the maximal therapeutic advantages to the patients)

→Thank you very much for your suggestion. We added it, please check again.

P13L23

In our hospital, opioid was started by oral administration of oxycodone and was switched to fentanyl tape when oral pain made it difficult for the patient to swallow. Switching to fentanyl tape was significantly lower among those patients who received PEMT versus those who did not, possibly because those procedures reduced their oral pain. Enforcement of correct mouthwash and moisturizer use by pharmacists enables the maximal therapeutic advantages to the patients.

Reviewer #2: The research is original, it has not been published nor the same data and analysis used elsewhere. The methodology and the statistical analysis were clarified. Also, the authors provide approval from their local area where the study was conducted.
In terms of the previous feedback, the authors have addressed the comments.
