# Supplementary material for: Pharmacist involved education program in a multidisciplinary team for oral mucositis: Its impact in head-and-neck cancer patients
Source: PLoS One. 2021 Nov 22;16(11):e0260026. doi: 10.1371/journal.pone.0260026 (PMC8608342; doi:10.1371/journal.pone.0260026)
Supplement: Attachment — Submitted filename: Reviewer comments (1).docx [file pone.0260026.s002.docx]

The paper documented the clinical outcomes of a pharmacist-involved education intervention among patients with head and neck cancer receiving concurrent chemoradiotherapy.

Major issues:

- There is potential of selection bias with a retrospective study design. For example, what are the selection criteria for a patient to be enrolled in the intervention compared to those without? The authors need to specify the efforts to reduce this bias. Although baseline characteristics of the two groups showed no statistical significance, but how were the number of study subjects determined and chosen? By inclusion of all and so-happened both groups were equal? Unless it is shown that selection bias is adequately addressed, the results and conclusion of the study may be invalid.
- An in-depth discussion of **how** a pharmacist helped in improving the clinical outcomes is required. Any documented increase in adherence to the prescribed regimen? It is suggested that more references to previous literatures should be included, especially in the field of oral care to substantiate the authors’ claims.

Minor issues:

- Duplicate keywords. Suggest ‘patient education’ to replace ‘education program’, ‘education’ and ‘advanced dental education’
- Please include statistical analysis in the abstract. Why 53 patients? Total samples during the stipulated timeframe?
- Please include study setting in the abstract
- p-value should not be capitalized
- Table 1: please double check the gender ratio under non-PEMT
- Table 1: weight loss should be an outcome rather than baseline characteristic
- Table 2: total sample was 53 but reduced to 47 in table 2?
- line 2 under introduction: amount
- Please provide the full name of the statistical software under methodology
- What are the criteria for PEMT compared to those without? If without PEMT, does it mean just without pharmacist (other healthcare professionals are available) or without a multidisciplinary team?
- If this was not the patients’ first cycle of chemotherapy, what was the history of OM occurrence between both comparison groups?
- 5^th^ paragraph of discussion: how had the involvement of a pharmacist reduced pain?
