# Supplementary material for: Pharmacist involved education program in a multidisciplinary team for oral mucositis: Its impact in head-and-neck cancer patients
Source: PLoS One. 2021 Nov 22;16(11):e0260026. doi: 10.1371/journal.pone.0260026 (PMC8608342; doi:10.1371/journal.pone.0260026)
Supplement: Attachment — Submitted filename: reviewer comments.docx [file pone.0260026.s004.docx]

Thank you very much for the efforts in the latest revision. The article is now more convincing and reads better.

Just a few minor suggestions as below:

1. Please double check the affiliation superscripts on the title page. What’s the difference between:
   a. ¶: These authors contributed equally to this work.
   b. &: These authors also contributed equally to this work.
2. Please ensure p-value is not capitalised in the main text and the figures
3. Please standardize to use either Figure or Fig.
4. Materials and methods> Subjects>
   a. Please capitalise ‘University Hospital’
   b. ‘the’ first cycle of chemotherapy
   c. >Definition of terms: please either define RT or use CCRT
5. Results> Opioids> line 2 should be (Table 3)
6. There are differences between values in Table 3 and in the result paragraph. Please double check.
7. Definition of RT is not needed in Table 1 since the term isn’t there.
8. Please remove the unnecessary ‘ ) ’ after a superscript annotation in all tables
9. Maybe can consider to the delete the intepretation of whisker plot?
   a. **Fig 3 Length of hospital days.** ~~a Data are shown in a box and whisker plot. The ends of the box represent the upper and lower quartiles and the median is depicted by the horizontal line within the box.~~
